# Identification of Aerococcus viridans in granulomatous mastitis utilizing nanopore targeted sequencing: case report and short literature review

**DOI:** 10.3389/fonc.2026.1735674

**Published:** 2026-05-13

**Authors:** Yingxiang Hui, Ping Zhao, Yue Gong, Yiyin Tang, Jiaqian Liao

**Affiliations:** 1 Department of Breast Surgery II, The Third Affiliated Hospital of Kunming Medical University, Kunming, Yunnan, China; 2 Department of Thoracic Surgery I, The Third Affiliated Hospital of Kunming Medical University, Kunming, Yunnan, China

**Keywords:** Aerococcus viridans, breast, clinic, granulomatous lobular mastitis, nanopore targeted sequencing

## Abstract

**Background:**

Granulomatous lobular mastitis (GLM) is a benign, nonspecific inflammatory disease associated with autoimmune dysregulation, pathogenic infection, endocrine disorders, and other etiological factors. Although various treatments for GLM exist, there is currently no standardized approach. *Aerococcus viridans* (*A. viridans*), an opportunistic pathogen typically non-pathogenic to humans, has been reported to cause infection in immunocompromised hosts. To date, there have been no documented cases of female granulomatous mastitis associated with *A. viridans*.

**Case description:**

A 33-year-old female presented with a right-breast mass. Ultrasound revealed multiple heterogeneous masses consistent with chronic GLM. Diseased glandular tissue was obtained under aseptic conditions for pathological examination and nanopore targeted sequencing (NTS). Pathology showed granulomatous inflammation, and NTS detected *A. viridans* as the dominant taxon.

**Conclusions:**

Granulomatous mastitis is a heterogeneous inflammatory condition in which pathogenic infection may contribute to disease development, although the spectrum of implicated microorganisms remains incompletely defined. *Aerococcus viridans* is rarely reported in this setting, and its detection in the present case broadens the range of microorganisms associated with GLM. The dominant detection of *A. viridans* by nanopore-targeted sequencing (NTS) indicates a potential association with the lesion; however, causality cannot be inferred from a single case. NTS is a valuable tool for rapid, accurate pathogen identification, supporting timely, rational antimicrobial decision-making. In this case, NTS-guided antibiotic therapy was followed by significant clinical improvement.

## Introduction

Granulomatous lobular mastitis (GLM), initially documented by Kessler and Wolloch in 1972 (1), is alternatively referred to as idiopathic granulomatous mastitis (IGM). It presents histopathologically as non-caseating granulomas predominantly localized within the lobules of the mammary gland in a multifocal pattern. This histological pattern is characterized by an increase in epithelioid cells, Langhans giant cells, neutrophils, as well as lymphocytosis (2). The incidence of GLM is reported to be highest within 5 years after lactation cessation (3), with patients typically around 30 years old (4). The condition’s prevalence is yet to be clearly defined, although a German study reported an annual prevalence of GLM of 2.4 per 100, 000 women aged 20 to 40 years (5). The underlying cause of the disease remains unclear, with various treatment methods available but no standardized approach. Some researchers suggest that the disease may have an autoimmune etiology, mainly involving immune cells and cytokines produced by immune cells, requiring hormone therapy for management (2). On the other hand, others propose that GLM may be associated with pathogenic infections (6), with symptomatic treatment supported by adequate antibiotics.

*Aerococcus viridans* (*A. viridans*), initially discovered by Williams in 1953 (7), is significant as an opportunistic pathogen that can be isolated from various specimens obtained from patients with compromised immunity and open trauma, including blood, urine, cerebrospinal fluid, and wound tissue fluid. Presently, specific prevalence data regarding this pathogen in humans and animals are lacking. Nonetheless, human diseases caused by *A. viridans* are infrequently reported. Yadav K et al. documented a case of severe endocarditis accompanied by aortic valve insufficiency resulting from *A. viridans* infection (8). Balvinder Mohan et al. presented two instances of nosocomial urinary tract infection caused by *A. viridans*, both confined to the urinary tract without bacteremia (9). Furthermore, A. Nasoodi et al. described a case of spondylodiscitis attributed to *A. viridans (*10). In India, Balvinder Mohan et al. reported a case of *A. viridans*-induced bacteremia, wherein Gram-positive cocci were identified in the blood culture smear and confirmed as *A. viridans*. Unfortunately, the patient succumbed to the *A. viridans* infection, likely due to advanced age and the presence of complications (11).

Nanopore-targeted sequencing (NTS) is a targeted sequencing approach that enables pathogen detection by analyzing bacterial 16S rRNA, fungal ITS1/2, and mycobacterial *rpoB* genes (12, 13). This report describes the detection of *A. viridans* in diseased glandular tissue from a patient with GLM using NTS.

## Case presentation

**Figure 1:**
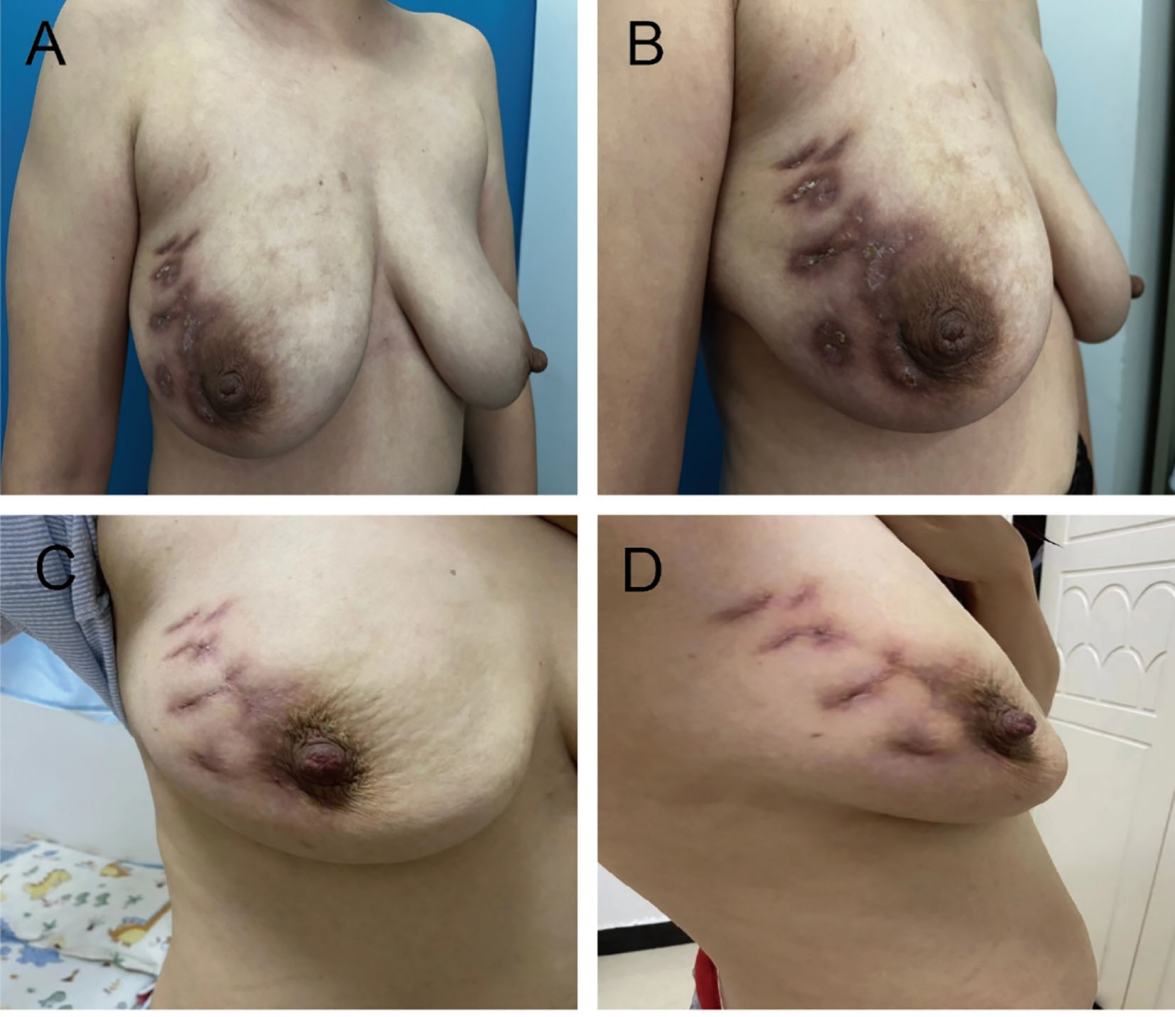
Clinical presentation and response to treatment. **(A, B)** Clinical appearance of the right breast at initial presentation, showing erythema, ulceration, multiple old scars, and pigmentation of the overlying skin. **(C, D)** Marked improvement after targeted antibiotic therapy, with resolution of skin erythema and swelling and complete healing of the ulceration.

A 33-year-old female patient presented to the hospital with complaints of a painful mass in her right breast. Prior to this, the patient had a history of chronic mastitis in the right breast, which persisted and worsened after multiple courses of antibiotics taken orally in an irregular manner. The patient had no history of hypertension, diabetes mellitus, autoimmune disorders, or any infections in other parts of the body. Her medical history included two pregnancies, one childbirth, and exclusive breastfeeding using breast milk for one year, with no reported incidents of milk blockage during lactation. The interval between the cessation of lactation and the onset of the disease was 2 years, between the onset of the disease and the last childbirth was 3 years, and between the onset of the disease and the last recurrence was 1 month. Physical examination revealed the presence of multiple palpable masses in the upper outer quadrant of the patient’s right breast, ranging in size from approximately 4.5 × 1.5 cm, fused and firm, with general mobility. The skin of the right breast appeared red and ulcerated, with multiple old scars, pigmentation, low skin temperature, no nipple depression, and no abnormal nipple secretions (**Figures 1A, B**). Related examination performed showed white blood cells (WBC) of 9.76×10^9^/L, absolute neutrophils(NEU) of 6.27×10^9^/L, absolute lymphocytes(LYM) of 2.96×10^9^/L, absolute monocytes of 0.36×10^9^/L, absolute eosinophils of 0.11×10^9^/L, absolute basophils (BAS) of 0.06×10^9^/L, C-reactive protein (CRP) of 1.37 mg/L, erythrocyte sedimentation rate (ESR) of 10 mm/h and prolactin(PRL) of 41.91 ng/mL. Color Doppler ultrasound showed multiple heterogeneous masses in the right breast. The larger one was located at 10 o’clock, about 41.2 × 11.67 mm in size, and characterized by a less clear boundary and an irregular shape, which were consistent with a diagnosis of chronic GLM. Under aseptic conditions, two portions of diseased glandular tissue were aspirated using fine needle aspiration into sterile sampling tubes for pathologic examination and NTS.

**Figure 2:**
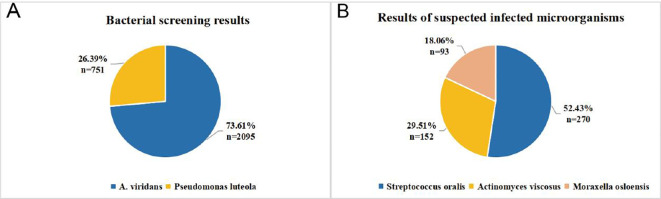
Nanopore-targeted sequencing (NTS) results. **(A)** Relative abundance of the dominant bacterial taxa detected by NTS. *Aerococcus viridans* accounted for 73.61% of total bacterial reads (n = 2, 095), followed by *Pseudomonas luteola* (26.39%, n = 751). **(B)** Bacterial taxa reported under the “suspected infection/commensal” category in the NTS report, including *Streptococcus oralis* (52.43%, n = 270), Actinomyces viscosus (29.51%, n = 152), and Moraxella osloensis (18.06%, n = 93).

**Figure 3:**
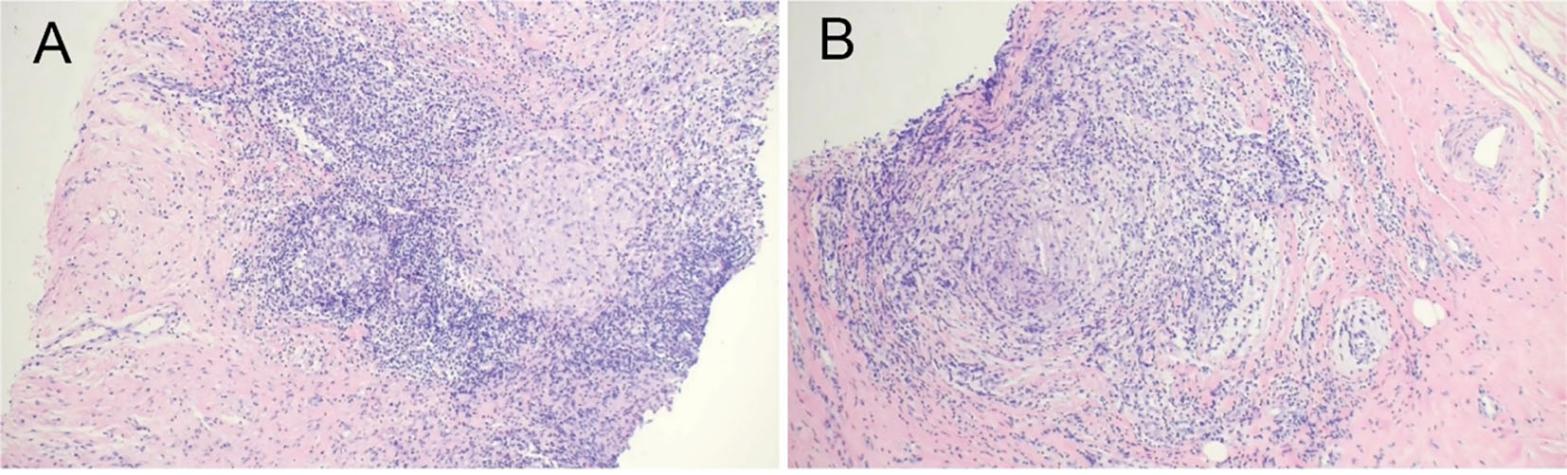
Histopathological findings of the breast lesion. **(A, B)** Hematoxylin and eosin–stained sections of the right breast lesion showing lobulocentric granulomatous inflammation with central suppuration, consistent with the histopathological features of granulomatous lobular mastitis (GLM) (original magnification ×100).

Nanopore-targeted sequencing (NTS) was performed using the Oxford Nanopore Technologies (ONT) MinION platform (R9.4.1 flow cell; standard ONT basecalling model, dna_r9.4.1_minion_384). The assay targets bacterial 16S rRNA, fungal ITS1/2, and mycobacterial *rpoB* genes for pathogen identification. NTS generated a total of 2, 846 bacterial reads, of which *Aerococcus viridans* accounted for 73.61% (n = 2, 095 reads; reported genome coverage, 97.51%) and *Pseudomonas luteola* for 26.39% (n = 751 reads; reported genome coverage, 98.82%); no fungi or viruses were detected. Additional taxa were reported under a “suspected infection/commensal” category, including *Streptococcus oralis* (n = 270), *Actinomyces viscosus* (n = 152), and *Moraxella osloensis* (n = 93). As noted in the NTS report, organisms in this category may represent environmental contamination, container-associated nucleic acids, or commensal microorganisms introduced during sampling (**Figures 2A, B**). The patient was diagnosed with granulomatous inflammation based on pathological examination (**Figures 3A, B**).

### DNA extraction and library preparation

DNA was extracted from diseased glandular tissue using the TIANamp Micro DNA Kit (Tiangen Biotech, Beijing, China) according to the manufacturer’s protocol. The DNA quality and concentration were assessed using a NanoDrop spectrophotometer (Thermo Fisher Scientific, Waltham, MA, USA). For library preparation, the Oxford Nanopore Ligation Sequencing Kit (SQK-LSK109, Oxford Nanopore Technologies, Oxford, UK) was used, and the procedure was performed according to the manufacturer’s instructions. A final library was prepared with an optimal DNA input for sequencing.

### Bioinformatic analysis

Nanopore-targeted sequencing (NTS) was performed using the Oxford Nanopore MinION platform (R9.4.1 flow cell; standard ONT basecalling model, dna_r9.4.1_minion_384). The assay targets bacterial 16S rRNA, fungal ITS1/2, and mycobacterial *rpoB* genes for pathogen identification. Basecalling was performed using the Guppy basecaller (version 3.3.0). Read filtering and sequence alignment were performed with Porechop (v.0.2.4) and BLASTn (v.2.9.0+), and the results were analyzed with Medaka (v.0.10.1) to generate consensus sequences. The microbial taxonomy assignment was performed using the SILVA database for 16S rRNA and ITS1/2 regions, and mycobacterial *rpoB* gene sequences were cross-referenced against the NCBI GenBank database.

Based on the NTS results, *A. viridans* and *Pseudomonas luteola* were considered the primary detected taxa. According to the antimicrobial susceptibility information provided with the report, both organisms were covered by piperacillin-tazobactam; therefore, piperacillin-tazobactam was selected. Intravenous piperacillin-tazobactam (4.5 g, twice daily) was administered for 2 weeks. On follow-up, breast redness and swelling subsided substantially, and the ulceration healed (**Figures 1C, D**). The patient reported resolution of pain and no palpable mass on examination.

## Discussion

The pathology of IGM involves the development of nodular lesions arising from the proliferation of local inflammatory macrophages and their derived cells, leading to a chronic inflammation accompanied by a delayed-type hypersensitivity reaction throughout its course (14, 15). The pathological findings of IGM indicate massive immune cell infiltration at the lesion site (16), with neutrophils surrounding Corynebacterium or other pathogens observed in granulomatous vacuoles (17), consistent with characteristics of chronic inflammation. The development of granulomatous chronic inflammation can be attributed to various factors, such as pathogens and foreign bodies (14, 15). Previous studies revealed the presence of pathogens and viruses in IGM tissues and pus, suggesting that pathogen infection may be a crucial factor in the development of IGM (18). Additionally, elevated WBC and CRP levels in blood indicators suggest pathogen infection (19). While not all patients exhibit standardized clinical manifestations and index changes, most present with changes at some stage of the disease.

Since 1985, studies have indicated that the positive rate of bacterial culture in nipple discharge caused by mammary duct dilatation is higher than other causes. This finding suggests a close association between the occurrence of this disease and the presence of pathogens (20). Corynebacterium has recently been recognized as the main flora responsible for granulomatous mastitis. In a study by Jiaxin Bi (21), corynebacterium was detected in up to 60% of cases. However, other species have also been identified with advancements in detection technology. For instance, Wang (22) detected the top five pathogens as Pseudomonas, Brevundimonas spp., Stenotrophomonas, Acinetobacter, and Aspergillus. Furthermore, another study (23) reported the detection of 24 species of bacteria, including Pallobacterium spp, Delftia spp, Streptococcus spp, and Doppleria spp. These findings suggest that granulomatous mastitis may be caused by a mixture of multiple bacterial infections rather than a single strain. Sequencing-based studies support this concept. Bi et al. demonstrated that GLM lesions harbor diverse bacterial communities using metagenomic next-generation sequencing, while other studies have similarly confirmed the polymicrobial nature of GLM (21). Corynebacterium species have been consistently associated with granulomatous mastitis (6).

Aerococcus species, including *A. viridans*, Aerococcus urinae (A. urinae), Aerococcus sanguinicola (A. sanguinicola), and Aerococcus suis (A. suis), are widely distributed in the air, soil, and hospital environments (24). *A. viridans* is a spherical bacterium measuring 1.0-2.0 μm in diameter and predominantly forms clusters and quadruplets in a liquid medium. It can exhibit alpha hemolysis on blood agar, characterized by a distinct grass-green hemolytic ring, thereby requiring differentiation from streptococcus species displaying similar hemolytic patterns (7). *A. viridans* is known to cause infections in both humans and animals, such as bovine mastitis, porcine arthritis, and porcine meningitis (25, 26). Although human infections are not frequently reported, there has been an increasing trend in recent years, particularly with regard to endocarditis (8) and urinary tract infections (9). Previous reports have described *A. viridans* infections in various organs, including infective endocarditis, urinary tract infections, and spondylodiscitis (27, 28). A comprehensive review by Rasmussen highlighted the emerging clinical relevance of Aerococcus species as opportunistic human pathogens (29).

*A. viridans* is a gram-positive coccus with known virulence features, including resistance to phagocytosis and the ability to adhere to host cells (30, 31). Additionally, previous studies have further demonstrated the pathogenic potential of Aerococcus viridans in mammalian models (32).

Generally, *A. viridans* is considered susceptible to beta-lactams, linezolid, and vancomycin, but resistant to levofloxacin (27). In our NTS results, *A. viridans* accounted for 73.61% of the bacterial reads, with *Pseudomonas luteola* accounting for the remaining 26.39%. Piperacillin-tazobactam, which provides activity against both organisms, was chosen in this case.

Routine culture, isolation, and biochemical reactions are the most commonly utilized methods for identifying microorganisms in clinical tests. However, using less common laboratory testing tools such as 16S rRNA sequencing and next-generation sequencing (NGS) is necessary for more accurate analysis and identification of some rare pathogens (33). In GLM, sequencing technologies like qPCR, Sanger sequencing, and NGS are employed for bacterial detection (22, 34), markedly enhancing the detection rate of microorganisms, with limited reports on the use of NTS in this setting. Previous studies have predominantly used formalin-fixed and paraffin-embedded (FFPE) tissues, which may result in DNA degradation. As a technology possessing numerous advantages, such as long read lengths (of up to 2.2 Mb), low data processing difficulty, rapidity and cost-effectiveness, and pocket-sized equipment (13), NTS can directly sequence entire DNA/RNA sequences, accurately identify species or finer branch species, and significantly improve diagnostic precision, particularly for patients with complex disease progression but without specific symptoms (12, 35). In a Japanese study that applied NTS to detect microorganisms in dairy cow milk, a vast number of strains were detected, with *A. viridans* constituting 10.4% (36). Qiong Huang et al. demonstrated the usefulness of NTS in identifying bacteria and fungi in patients with infectious endophthalmitis, with the test results consistent with microbial culture findings (35). In Their study, the average waiting time for bacterial and fungal culture results was 48 hours and 72 hours, respectively. In comparison, the average waiting time for NTS results was 12 hours, indicating NTS’s accuracy and speed advantages.

To date, *A. viridans* has rarely been reported in breast-related infections, and no prior studies have clearly documented its presence in granulomatous lobular mastitis. In contrast, previous studies have mainly identified Corynebacterium species or mixed microbial communities in GLM lesions, likely reflecting the limitations of conventional culture-based methods in detecting fastidious or low-abundance organisms. The present case expands the microbial spectrum of GLM and a potential association with rare or opportunistic pathogens such as *A. viridans*. Nanopore-targeted sequencing enabled rapid and accurate pathogen identification, supporting its value in guiding individualized antimicrobial therapy.

This report is based on a single patient, and the detection of *A. viridans* should not be interpreted as evidence of a causal role in granulomatous lobular mastitis (GLM). Multiple bacterial taxa were identified, and sequence-based methods alone cannot clearly distinguish actual infection from colonization or potential contamination. Because no blank or negative controls were processed in parallel, laboratory contamination cannot be entirely excluded. Clinical improvement occurred after administration of a broad-spectrum antimicrobial regimen active against multiple organisms; therefore, the therapeutic response cannot be specifically attributed to *A. viridans*. The patient had no clinical features suggestive of systemic infection, such as fever or bacteremia; however, because blood cultures were unavailable, systemic infection cannot be completely excluded. Moreover, confirmatory investigations such as culture, targeted PCR, repeat sampling, negative controls, or tissue-based localization were not performed in this case. Despite these limitations, *A. viridans* showed a dominant read count and broad reported genome coverage, suggesting that its detection was unlikely to reflect a sporadic or low-level background signal. Further studies in larger GLM cohorts, combining sequencing results with conventional microbiological and pathological confirmation, may help clarify the clinical relevance of Aerococcus species and better define their role in guiding individualized treatment strategies.

## Data Availability

The data presented in this study are available from the corresponding author upon reasonable request.
